# A tsRNA Suppresses Lung Adenocarcinoma Progression Through Targeting Glucose‐6‐Phosphate Dehydrogenase

**DOI:** 10.1002/jbt.71077

**Published:** 2026-09-02

**Authors:** Suo Sha, Meilin Zhang, Qinsong Tao, Yang Liu, Junjun Bao, Ping Guo, Aiting Yan, Cuizhu Wang

**Affiliations:** ^1^ Surgery of Traditional Chinese Medicine, Hai'an Hospital of Traditional Chinese Medicine Hai'an, Nantong Jiangsu Province China; ^2^ Department of Hematology and Oncology Hai'an City People's Hospital of Jiangsu Province Hai'an, Nantong Jiangsu Province China

## Abstract

Elucidating the molecular distinctions between invasive and non‐invasive forms of lung adenocarcinoma is essential for the development of personalized therapeutic approaches and the optimization of patient outcomes. Transfer RNA‐derived small RNAs (tsRNAs) have been recognized as pivotal regulators in oncogenic processes. In this study, we identified a specific tsRNA associated with non‐invasive lung adenocarcinoma and demonstrated its suppressive effects on cellular proliferation and metastatic potential. At the molecular level, this tsRNA exerts its regulatory function by targeting and repressing G6PD mRNA, thereby perturbing the equilibrium between NADPH and reactive oxygen species (ROS) production. This discovery positions the identified tsRNA as a promising candidate for therapeutic intervention in non‐invasive lung adenocarcinoma. Further investigation into the role of tsRNAs in lung adenocarcinoma is imperative to fully harness their potential in clinical management.

## Highlights

- tsRNA‐Thr‐5‐0096 is significantly down‐regulated in invasive LUAD tissues and promotes LUAD cells metastasis and growth.
- tsRNA‐Thr‐5‐0096 suppresses LUAD progression by targeting G6PD which regulates glycolysis and oxidative stress.
- G6PD mediated the function of tsRNA‐Thr‐5‐0096 on LUAD progression.

## Graphical abstract

Structure of tsRNA‐Thr‐5‐0096. Mechanically, tsRNA‐Thr‐5‐0096 suppresses LUAD progression by targeting G6PD which regulates glycolysis and oxidative stress.

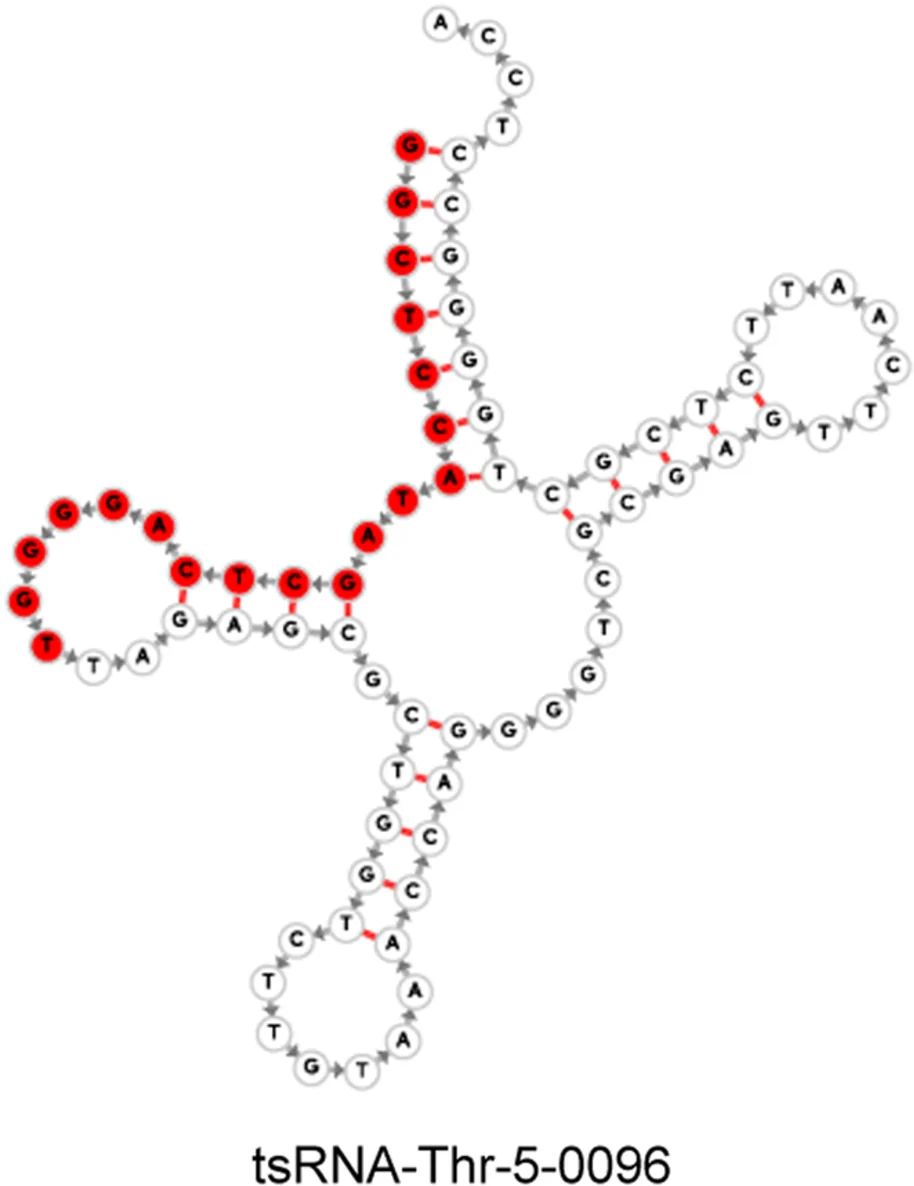

## 1 Introduction

Lung cancer continues to be a leading cause of cancer‐related mortality globally [1], largely due to its propensity for metastasis [2]. Among the various subtypes, lung adenocarcinoma represents the most prevalent form. Invasive and non‐invasive lung adenocarcinoma are prominent subtypes of lung cancer, which differ in terms of metastatic tendencies, diagnosis, and treatment approaches [3, 4]. Invasive lung adenocarcinoma exhibits higher invasiveness and metastatic potential, while non‐invasive lung adenocarcinoma tends to remain localized. However, the molecular mechanisms between invasive and non‐invasive lung adenocarcinoma remain poorly understood, which is crucial for developing personalized treatment strategies and improving patient outcomes.

Transfer RNA‐derived small RNA molecules (tsRNAs) have traditionally been known for their indispensable role in protein synthesis [5]. However, accumulating evidence suggests that tsRNAs play diverse roles beyond translation and are implicated in various physiological and pathological processes, including cancer [6, 7, 8, 9, 10]. Particularly, aberrant expression of tsRNAs has been observed across numerous cancer types, highlighting their potential as diagnostic and prognostic biomarkers [11, 12]. The functional mechanisms by which tsRNAs contribute to cancer development are still being elucidated. It has been suggested that tsRNAs can modulate gene expression by directly interacting with messenger RNAs (mRNAs) or by influencing the activity of RNA‐binding proteins [5]. Additionally, tsRNAs have been implicated in regulating the activity of microRNAs (miRNAs), another class of ncRNAs involved in post‐transcriptional gene regulation [13, 14]. However, few studies focused on the role of tsRNAs in LUAD.

In this study, we identified a tsRNA associated with non‐invasive lung adenocarcinoma. In vitro functional assays have demonstrated that this tsRNA inhibits the proliferation and metastasis of lung adenocarcinoma cell lines. In terms of mechanism, we found that tsRNA inhibits the mRNA expression of G6PD through a type of miRNA‐like manner, thereby disrupting the balance between NADPH and ROS, leading to inhibition of the malignant progression of lung adenocarcinoma. Overall, this tsRNA may serve as a novel and effective therapeutic molecular target for lung adenocarcinoma.

## 2 Methods

### 2.1 Clinical Samples

6 LUAD tissue samples, among which 3 cases were pathologically diagnosed as invasive lung adenocarcinoma (invasive adenocarcinoma, IA) and 3 cases were non‐invasive lung adenocarcinoma (adenocarcinoma in situ, AIS/microinvasive adenocarcinoma, MIA), were used for tRFs & tiRNAs sequencing. We randomly selected 40 samples of invasive lung adenocarcinoma (LUAD) tissues and non‐invasive LUAD tissues, which were obtained from patients who underwent lobectomy resection and were diagnosed with LUAD at Jiangsu Cancer Hospital between 2018 and 2021. Clinical information is provided in Supporting Information S2: Table S1. Both tissue and peripheral blood samples were collected from the Jiangsu Cancer Hospital Biobank. This study was conducted in accordance with the ethical principles outlined in the Declaration of Helsinki. Approval for this study was obtained from the Ethics Committee of the Affiliated Cancer Hospital of Nanjing Medical University, and written consent was obtained from all participating patients.

### 2.2 Cell Lines

The following human lung adenocarcinoma (LUAD) cell lines were obtained from the cell bank of the Shanghai Institute of Cell Biology (Chinese Academy of Medical Science, Shanghai, China): A549, PC9, HCC827, H1975, and the human bronchial epithelium cell line HBE. These cell lines were cultured in RPMI‐1640 complete medium or Dulbecco's modified Eagle's medium (KeyGEN, Nanjing, China), supplemented with 10% fetal bovine serum (FBS, Gibco, NY, USA) and 1% penicillin‐streptomycin (Gibco). Cultures were maintained in a 5% CO2 incubator at 37°C.

### 2.3 Animal Model

Six‐ to 8‐week‐old male C57BL/6 J mice were housed under SPF conditions. Tranfected LLC1 cells were cultured in DMEM supplemented with 10% FBS and antibiotics, harvested in the logarithmic phase, washed, and resuspended in PBS at 1 × 10^7^ cells/mL. A single‐cell suspension (1 × 10^6^ cells in 100 µL PBS) was injected subcutaneously into the right flank of each mouse. Tumor dimensions were measured with a digital caliper every 2–3 days, and volume was calculated as 0.5 × (long diameter) × (short diameter)^2^.

### 2.4 Subcellular Fractionation

For the detection of tsRNA‐Thr‐5‐0096 expression in cytoplasmic and nuclear fractions, the PARIS protein and RNA Isolation Kit (Invitrogen) was used according to the manufacturer's instructions. RNA was extracted from cytoplasmic and nuclear fractions and subjected to quantitative PCR (qPCR). Nuclear and cytoplasmic markers utilized were U6 and GAPDH, respectively.

### 2.5 RNA Extraction and qRT‐PCR

Total RNA was extracted from both tissue samples and cell lines using TRIzol reagent (Invitrogen, USA) following the manufacturer's protocol. qRT‐PCR was performed with the SYBR Green Master Mix (Vazyme Biotech). The 2^−ΔΔCt^ method was used for data analysis, with β‐actin or U6 as the internal control. The StepOnePlus Real‐Time PCR system (Applied Biosystems, USA) was employed for data collection. For tsRNA quantification, Bulge‐LoopTM MicroRNA qRT‐PCR Primer Sets were specifically designed. Other primer information is as follows:

U6 RNA forward, 5′‐TTATGGGTCCTAGCCTGAC‐3′ and reverse, 5′‐CACTATTGCGGGTCTGC‐3′; G6PD forward, 5′‐AACATCGCCTGCGTTATCCTC‐3′ and reverse, 5′‐ACGTCCCGGATGATCCCAA‐3′.

### 2.6 Fluorescence in Situ Hybridization (FISH)

Sections of A549 or PC9 cells (1 × 10^5^) were fixed in 4% paraformaldehyde for 10 min and washed with PBS. Permeabilization was performed using 0.5% Triton X‐100 in pre‐cooled PBS at 4°C for 15 min. The cells were then subjected to a 4‐h incubation at 37°C with a mixture of cy3‐labeled tsRNA‐Thr‐5‐0096 probe (5′−3′) (Supporting Information S2: Table S2). Probe signals were detected using a FISH kit (GenePharma China) according to the manufacturer's instructions. DAPI was used for nuclear staining. Confocal microscopy (Carl Zeiss, Germany) was used to capture the images.

### 2.7 Cell Transfection

The tsRNA‐Thr‐5‐0096 inhibitor was obtained from Genepharm (Shanghai, China), while the tsRNA‐Thr‐5‐0096 mimic was provided by RiboBio. Transfection was performed using Lipofectamine iMAX (Thermo, USA). The G6PD cDNA was synthesized from Invitrogen and cloned into the expression vector pcDNA3.1. Lipofectamine 3000 (Thermo, USA) was used for transfection following the manufacturer's instructions. The nucleotide sequences and concentrations are shown in Supporting Information S2: Table S2.

### 2.8 5‐ethynyl‐2'‐Deoxyuridine (Edu) Assay

A549 and PC9 cells were seeded in 96‐well plates, harvested 48 h later, and then incubated with 50 mM EdU for 2 h. Fixed with 4% paraformaldehyde, the cells were incubated with Apollo Dye Solution to label proliferating cells. DAPI was used for nuclear counterstaining. The green signal of proliferating cells was visualized using a microscope (Carl Zeiss, Germany).

### 2.9 Cell Proliferation Assay and Colony Formation Assay

A certain number (5 × 10^3^) of transfected A549 or PC9 cells were seeded in a 96‐well plate. After incubation for 2, 24, 48, 72, and 96 h, 10 μL of CCK8 solution (Dozu Island, Japan) was added to each well, and the OD value at 450 nm was measured using a microplate reader.

### 2.10 Wound Healing Assay

LUAD cells were seeded in 6‐well plates and transfected. After 24 h of transfection, the cell monolayers were wounded by scratching with a sterile 1000‐μL micropipette tip. Immediately and 36 h after wounding, the cells were imaged using phase‐contrast microscopy.

### 2.11 Glucose Uptake, Lactate Production, and ATP Production Assay

The glucose uptake assay was performed using the Glucose Uptake Fluorometric Assay Kit (MAK084, Sigma‐Aldrich) following the manufacturer's instructions. The lactate production assay was carried out using the Lactic Acid assay kit (KGT023, KeyGEN Biotech) as per the manufacturer's protocol. ATP production was determined using the Enhanced ATP Assay Kit (S0027, Beyotime) following the manufacturer's protocol. The result was normalized to total protein.

### 2.12 NADPH and ROS Production Assay

For the NADPH and ROS production assay, the NADPH and ROS levels were measured using the NADP + /NADPH detection kit (S0179, Beyotime) and the Reactive Oxygen Species Assay Kit (S0033S, Beyotime), respectively, according to the manufacturer's instructions. The result was normalized to total protein.

### 2.13 JC‐1 and ROS Staining

LUAD cells were seeded at an appropriate density and treated as indicated. Following treatment, cells were incubated with JC‐1 (10 μg/mL) or DCFH‐DA (10 μM) in serum‐free medium at 37°C for 20 min and 30 min, respectively, protected from light. After washing three times with PBS, fluorescence signals were immediately detected by flow cytometry or fluorescence microscopy. Mitochondrial membrane potential was expressed as the ratio of red to green fluorescence, and intracellular ROS levels were quantified by relative DCF fluorescence intensity normalized to the control group.

### 2.14 Oxygen Consumption Rate (OCR) Quantification

To quantify the oxygen consumption rate (OCR), OCR in A549 cells was measured using an extracellular flux analyzer (XFe24 Seahorse; Agilent Technologies) following the manufacturer's protocol for mitochondrial stress tests. The measurement cycles consisted of 1.5 min of mixing, 2 min of waiting, and 2 min of measuring. Basal OCR was measured for 3 cycles, while other conditions were measured for 2 cycles. The concentrations of drugs used were oligomycin (1 μM, Sigma, 75351), oligo cyanide‐4‐(trifluoromethoxy) phenylhydrazone (FCCP) (2 μM Sigma, C2920), rotenone (1 μM, Sigma, A8674), and antimycin A (1 μM, Sigma, R8875). After all measurements, the cells were fixed with 2% paraformaldehyde for 10 min at room temperature and incubated with DRAQ5™ (Thermo Fisher) (1:1000) for 20 min at room temperature.

### 2.15 Western Blot

For the Western blot analysis, cells were washed with PBS and lysed on ice using a mixture of RIPA (#89901, Thermo) and protease inhibitors (#A32963, Thermo). The lysates were centrifuged, and SDS‐PAGE loading buffer (#P0015F, Beyotime) was added at a 1:5 ratio to prepare protein samples by heat denaturation. The protein concentration was determined using the BCA Protein Assay kit (#P0010S, Beyotime). Protein samples were separated by electrophoresis on a 10% SDS‐PAGE gel, transferred to PVDF membranes, blocked with skim milk, and incubated with primary antibodies overnight. The primary antibodies used were anti‐G6PD (#15071S, Cell Signaling) and anti‐GAPDH (#5174S, Cell Signaling). Fluorescently labeled secondary antibodies (#611‐145‐002 & #610‐144‐002, Rockland) were applied for 1.5 h. Finally, the PVDF membrane was detected using the Odyssey Clx (LICOR) detector.

### 2.16 Dual Luciferase Reporter Assay

The G6PD miR‐RNA binding sites were predicted using TargetScan (http://www.targetscan.org). The sequence interacting with the seed sequence of tsRNA was mutated, and the wild‐type and mutant G6PD 3′‐UTR were inserted into the pGL3 basic vector (Promega) to test for binding specificity. All vectors were validated by sequencing, and luciferase activity was assessed using the Dual Luciferase Assay kit (Promega) following the manufacturer's instructions.

### 2.17 Statistical Analysis

GraphPad Prism 9.4 software was used for statistical analysis. Most plots contain plots for each data point and show the mean ± standard deviation. The t‐test was used to test for significance, and *P* values are indicated by asterisks. The *P* value of the CCK‐8 assay was determined by two‐way ANOVA with Tukey's multiple comparison test.

## 3 Results

### 3.1 tsRNA‐Thr‐5‐0096 Was Significantly Down‐Regulated in Invasive LUAD Tissues

**Figure 1:**
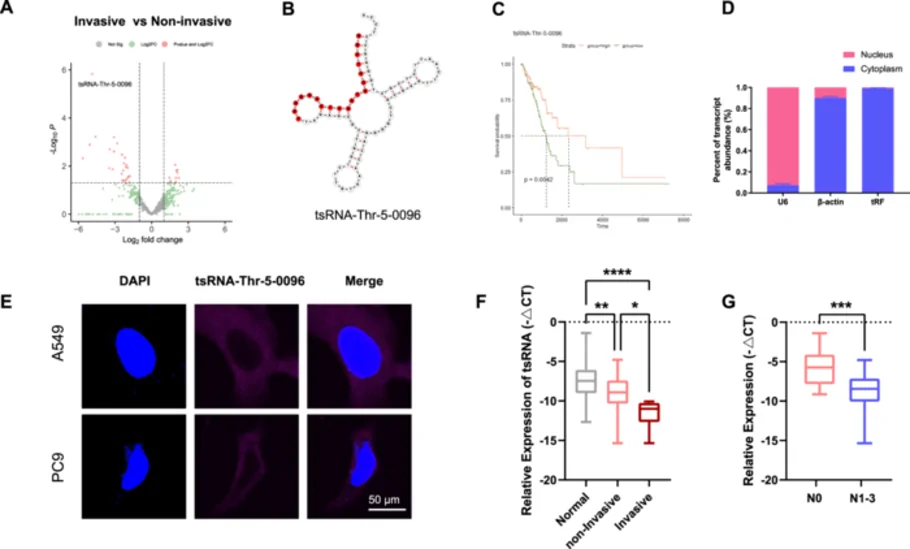
The down‐regulation of tsRNA‐Thr‐5‐0096 in invasive LUAD tissues**.** (A) tRFs & tiRNAs sequencing was conducted on 3 invasive lung adenocarcinoma tissues and 3 non‐invasive lung adenocarcinoma tissues, followed by further analysis of the results. (B) A pattern diagram illustrating the sequence of tsRNA‐Thr‐5‐0096. (C) Kaplan‐Meier survival analysis of LUAD patients based on higher or lower levels of tsRNA‐Thr‐5‐0096 in the TCGA database. (D) The expression of tsRNA‐Thr‐5‐0096 in the A549 cell line was analyzed using qRT‐PCR. (E) FISH assays visualized the location of tsRNA‐Thr‐5‐0096 in A549 and PC9 cells, with a scale bar of 50 μm. (F) The expression of tsRNA‐Thr‐5‐0096 in invasive and non‐invasive LUAD tissues, as well as the paired normal tissues were determined by qRT‐PCR (*n* = 40). (G) The expression of tsRNA‐Thr‐5‐0096 in N0 and N1‐3 LUAD tissues was analyzed by qRT‐PCR.

To identify potential dysregulated tsRNAs between invasive LUAD tissues (invasive adenocarcinoma, IA) and non‐invasive LUAD tissues (adenocarcinoma in situ, AIS/microinvasive adenocarcinoma, MIA), 6 LUAD tissue samples, among which 3 cases were pathologically diagnosed as invasive lung adenocarcinoma, and 3 cases were non‐invasive lung adenocarcinoma, were used for tRFs & tiRNAs sequencing. (Figure 1A). The results showed that 25 tsRNAs were decreased and 9 tsRNAs were increased, respectively (Figure 1A). The tsRNA‐Thr‐5‐0096 (19 nt, derived from 5′ tRNA‐Thr‐TGT‐3‐1) was down‐regulated most significantly among the 34 dysregulated tsRNAs, which indicated that tsRNA‐Thr‐5‐0096 may be associated with non‐invasive LUAD tissues (Figure 1B). Furthermore, results showed tsRNA‐Thr‐5‐0096 was associated with overall survival in LUAD patients in the TCGA database (Figure 1C).

To verify the distribution of tsRNA‐Thr‐5‐0096 in LUAD cells, a specific fluorescent probe for tsRNA‐Thr‐5‐0096 was designed, which showed that tsRNA‐Thr‐5‐0096 was mainly distributed in the cytoplasm of LUAD cells, consistent with the results of RT‐qPCR (Figure 1D,E). The expression levels of tsRNA‐Thr‐5‐0096 in 40 invasive LUAD tissues and 40 non‐invasive LUAD tissues were also detected by qPCR. Interestingly, tsRNA‐Thr‐5‐0096 was significantly downregulated in invasive LUAD tissues compared with non‐invasive LUAD tissues (Figure 1F). Moreover, tsRNA‐Thr‐5‐0096 expression was significantly lower in LUAD tissues with higher N stage (Figure 1G). These data suggest that tsRNA‐Thr‐5‐0096 may be a potential therapeutic target in the malignant progression of LUAD.

### 3.2 tsRNA‐Thr‐5‐0096 Promotes LUAD Cells Metastasis and Growth

**Figure 2:**
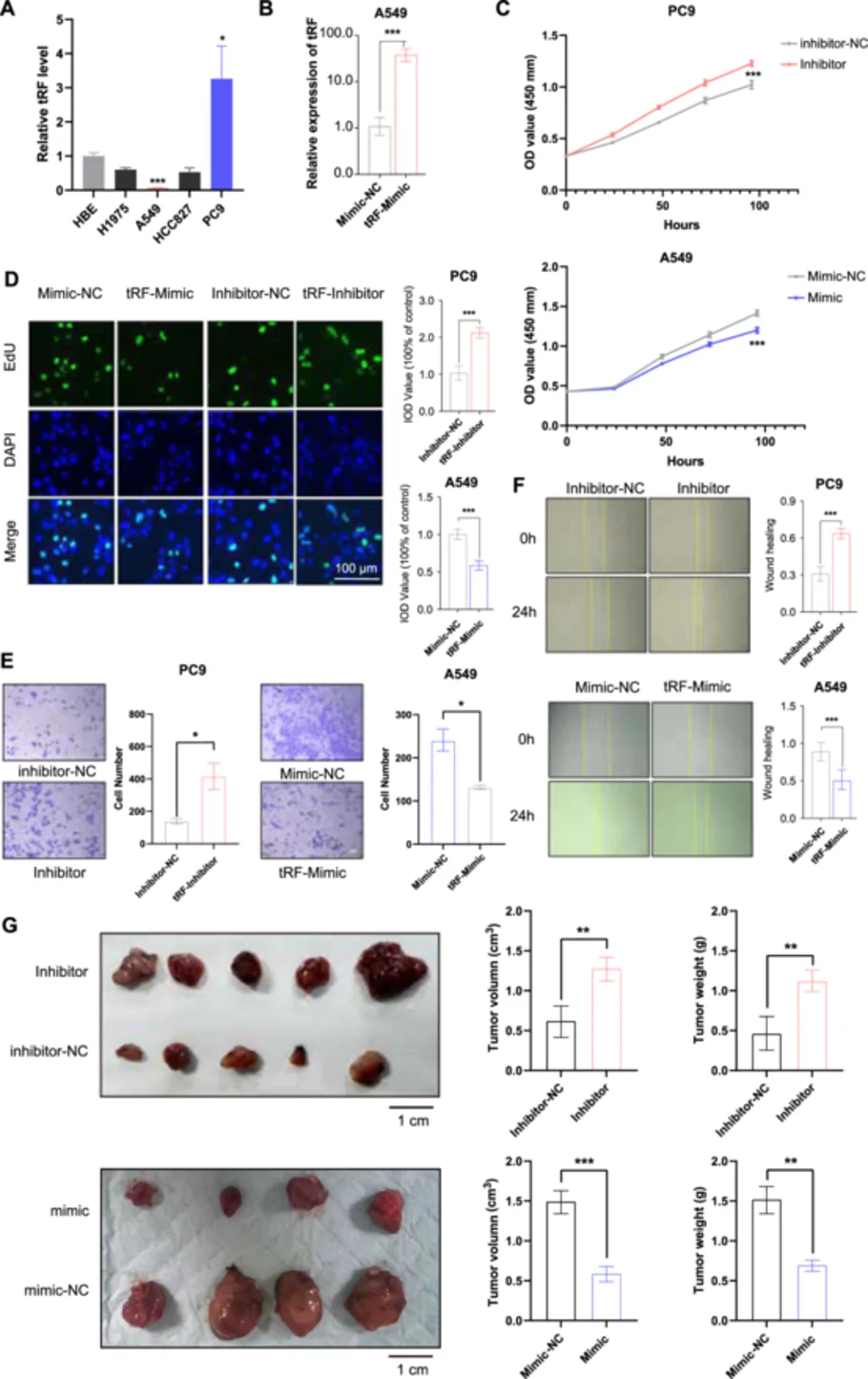
tsRNA‐Thr‐5‐0096 promotes LUAD cells metastasis and growth. (A) The expression of tsRNA‐Thr‐5‐0096 in various LUAD cell lines. (B) The transfection efficiency of tRF‐mimic was confirmed using qRT‐PCR. (C) CCK‐8 assays were performed on A549 and PC9 cells transfected with tRF‐mimic, mimic‐NC, inhibitor, or inhibitor‐NC. (D) EdU assay was conducted on A549 and PC9 cells transfected with tRF‐mimic, mimic‐NC, inhibitor, or inhibitor‐NC, with a scale bar of 100 μm. (E) Transwell assay was performed on A549 and PC9 cells transfected with tRF‐mimic, mimic‐NC, inhibitor, or inhibitor‐NC, with a scale bar of 50 μm. (F) Wound healing assay was conducted on A549 and PC9 cells transfected with tRF‐mimic, mimic‐NC, inhibitor, or inhibitor‐NC, with a scale bar of 50 μm. (G) The tumor‐bearing assay using inhibitor or inhibitor‐NC, mimic or mimic‐NC transfected LLC1 cells in vivo.

tsRNA‐Thr‐5‐0096 mimic and inhibitor were selected for overexpression and inhibition. The expression of tsRNA‐Thr‐5‐0096 in LUAD cell lines was validated (Figure 2A). The expression of tsRNA‐Thr‐5‐0096 in LUAD cell lines was validated, and transfection efficiency was evaluated using qRT‐PCR (Figure 2B). Due to the competitive binding mechanism of the inhibitor, the transfection efficiency was not validated by qRT‐PCR. Gain‐ and loss‐of‐function experiments were conducted in vitro to investigate the biological function of tsRNA‐Thr‐5‐0096 in LUAD malignant progression.

EdU assays and CCK‐8 assays were performed, and it was identified that overexpression of tsRNA‐Thr‐5‐0096 significantly promoted cell proliferation of the LUAD cell lines, whereas inhibition of tsRNA‐Thr‐5‐0096 significantly suppressed LUAD cell lines' proliferation (Figure 2C,D). Furthermore, wound healing and transwell assays indicated that tsRNA‐Thr‐5‐0096 substantially decreased LUAD cell migration and invasion (Figure 2E,F). For further validation of the function of tsRNA‐Thr‐5‐0096 in vivo, we further established a tumor‐bearing model using inhibitor, inhibitor‐NC, mimic, or mimic‐NC‐transfected LUAD cells. The result indicated that the tumors in the inhibitor group grew significantly faster than the inhibitor‐NC group, with higher weight and larger volume (Figure 2G) and vice versa.

### 3.3 tsRNA‐Thr‐5‐0096 Suppresses LUAD Progression by Targeting G6PD

**Figure 3:**
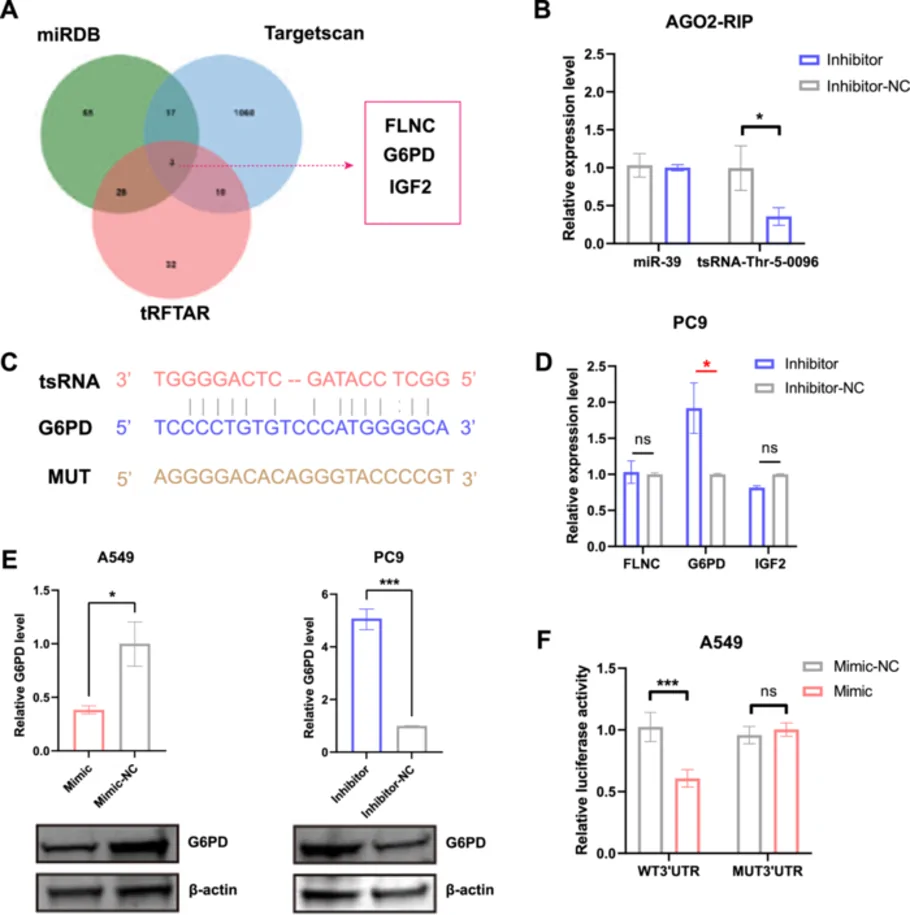
tsRNA‐Thr‐5‐0096 suppresses LUAD progression by targeting G6PD. (A) Venn diagram displaying the potential downstream targets of tsRNA‐Thr‐5‐0096 based on three online databases: TargetScan, miRDB, and tRFexplorer. (B) AGO2‐RIP assay confirmed the interaction between tsRNA‐Thr‐5‐0096 and AGO2 protein in A549 cells. (C) Upper: Pattern diagram illustrating the complementary pairing between tsRNA‐Thr‐5‐0096 and partial sequences of G6PD 3′ UTR. Lower: Schematic representation of the luciferase plasmid design for wild‐type and mutant forms. (D) qRT‐PCR analysis of Filamin C (FLNC), G6PD, and IGF‐2 mRNA levels in PC9 cells transfected with inhibitior‐NC and inhibitor. (E) qRT‐PCR and WB analysis of G6PD mRNA and protein levels in A549 and PC9 cells transfected with tRF‐mimic, mimic‐NC, inhibitor, or inhibitor‐NC. (F) Dual‐luciferase reporter assay examining the direct binding between G6PD 3′ UTR and tsRNA‐Thr‐5‐0096.

Previous studies have found that tsRNAs may play an important role in tumor progression through a miRNA‐like manner [15, 16, 17]. To explore the downstream target of tsRNA‐Thr‐5‐0096, the potential target genes of tsRNA‐Thr‐5‐0096 were also predicted by the intersection of 3 online databases: TargetScan (https://www.targetscan.org/vert_72/), miRDB (https://mirdb.org/), and tRFTAR (https://www.rnanut.net/tRFTar/#) (Figure 3A). Due to the fact that miRNA‐like effects of tsRNAs are often caused by tsRNA binding to AGO2 protein and then targeting the 3′UTR region of mRNA, we conducted AGO2‐RIP experimental analysis and found that AGO2 can bind to tsRNA‐Thr‐5‐0096 (Figure 3B), indicating that tsRNA‐Thr‐5‐0096 may regulate LUAD progression through a miRNA‐like manner. Filamin C (FLNC), glucose‐6‐phosphate dehydrogenase (G6PD), and IGF2 were screened through bioinformation analysis databases. Through validation by qRT‐PCR, only G6PD mRNA level was significantly decreased after tsRNA‐Thr‐5‐0096 overexpression, suggesting that G6PD may be a downstream target of tsRNA‐Thr‐5‐0096 (Figure 3D). And the target site was predicted using TargetScan (https://www.targetscan.org/vert_72/)(Figure 3C). To further confirm that the regulatory role of tsRNA‐Thr‐5‐0096 on G6PD, we examined the change of G6PD mRNA and protein levels after inhibition or overexpression of tsRNA‐Thr‐5‐0096, which showed that the protein level of G6PD was significantly reduced after tsRNA‐Thr‐5‐0096 overexpression and vice versa (Figure 3E). Meanwhile, the expression of tsRNA‐Thr‐5‐0096 is higher in PC9 than in A549. We performed a transwell assay on the two cell lines, and the result indicated that the invasive ability of A549 is significantly higher than that of PC9 (Supporting Information S1: Figure S1A). Subsequently, dual‐luciferase reporter assay showed decreased luciferase activity with transfection of G6PD 3′ UTR wild‐type reporter plasmid, not with the mutant G6PD plasmid. (Figure 3F). These results indicate that tsRNA‐Thr‐5‐0096 suppresses LUAD progression by targeting G6PD.

### 3.4 G6PD Promotes LUAD Progression by Regulating Glycolysis and Oxidative Stress

**Figure 4:**
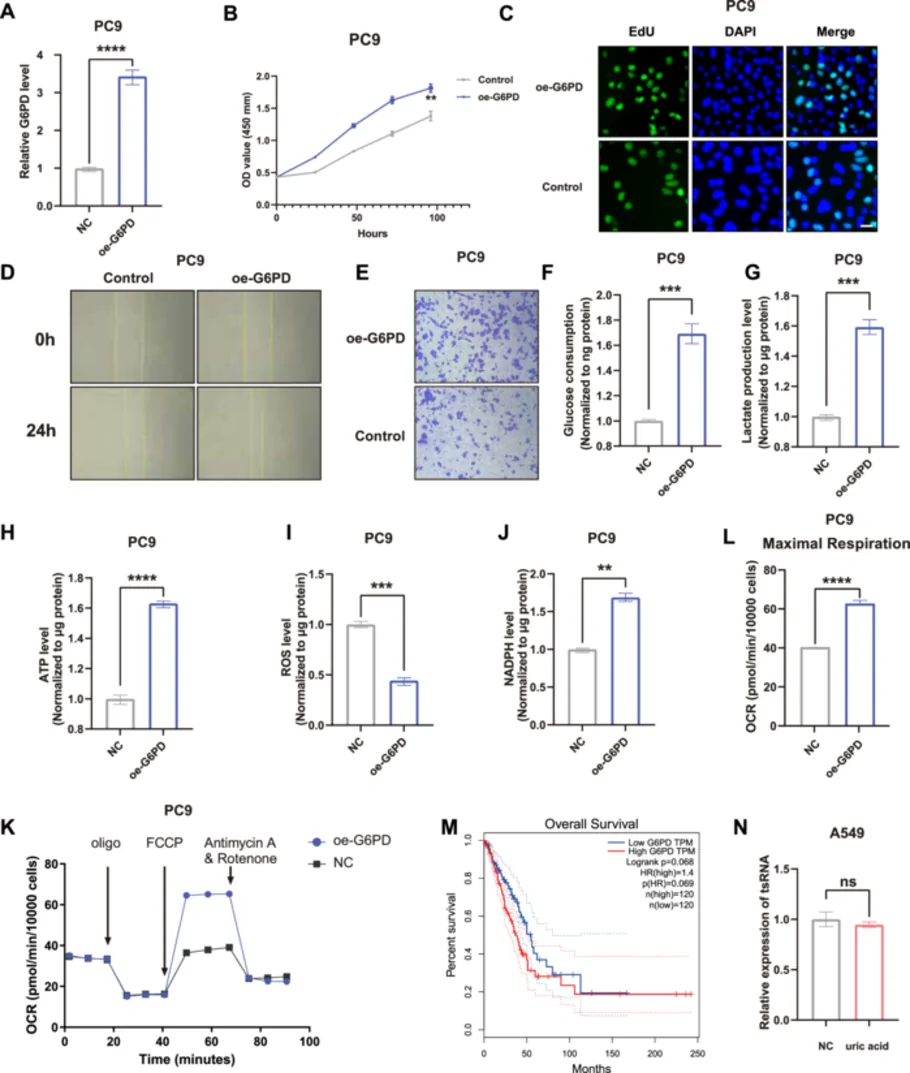
G6PD promotes LUAD progression by regulating glycolysis and oxidative stress. (A) qRT‐PCR confirmed the transfection efficiency of oe‐G6PD. (B) CCK‐8 assays were conducted on PC9 cells transfected with oe‐G6PD or oe‐NC. (C) EdU assay was performed on PC9 cells transfected with oe‐G6PD or oe‐NC, with a scale bar of 100 μm. (D) Transwell assay was conducted on PC9 cells transfected with oe‐G6PD or oe‐NC, with a scale bar of 50 μm. (E) Wound healing assay was performed on PC9 cells transfected with oe‐G6PD or oe‐NC, with a scale bar of 50 μm. (F–H) Measurement of glucose uptake (F), lactate production (G), and ATP levels (H) in PC9 cells transfected with oe‐G6PD or oe‐NC. (I, J) Measurement of NADPH level (I) and ROS level (J) in PC9 cells transfected with oe‐G6PD or oe‐NC. (K, L) OCR measurement in PC9 cells transfected with oe‐G6PD or oe‐NC. (M) Survival analysis based on G6PD expression was performed in GEPIA (gepia. cancer‐pku. cn). (N) qRT‐PCR analysis of tsRNA‐Thr‐5‐0096 in uric acid‐treated A549 cells.

In previous studies, it has been reported that G6PD can promote cancer progression. Then we validated its role in lung adenocarcinoma cells. The plasmid of oe‐G6PD was transfected into lung adenocarcinoma cell lines and its efficiency was verified by qPCR (Figure 4A, Supporting Information S1: Figure S1B), followed by in vitro functional experiments. EdU assays and CCK‐8 assays were performed, and it was identified that overexpression of G6PD significantly promoted cell proliferation of the LUAD cell lines. (Figure 4B,C, Supporting Information S1: Figure S1C,D). Furthermore, the migration and invasion abilities of LUAD cell lines were detected by wound healing and transwell assays, which suggested that overexpression of G6PD could significantly promote LUAD cell migration and invasion (Figure 4D,E, Supporting Information S1: Figure S1E). G6PD catalyzes the rate‐limiting step of the pentose phosphate pathway (PPP), which is responsible for generating NADPH and is involved in the protection against oxidative stress. Additionally, as previously reported, G6PD was also associated with glycolysis. Our results showed that overexpression of G6PD notably enhanced glucose utilization, lactate concentrations, and ATP production in A549 cells (Figure 4F–H, Supporting Information S1: Figure S1F–H). Furthermore, overexpression of G6PD increased NADPH production while reducing ROS levels (Figure 4I,J, Supporting Information S1: Figure S1I,J). In addition, OCR experiments have demonstrated that overexpression of G6PD enhances maximal respiration in A549 cell lines (Figure 4K,L, Supporting Information S1: Figure S1K,L). These data indicated that G6PD could promote LUAD progression through regulating glycolysis and oxidative stress. As shown in the GEPIA database, patients with high G6PD levels seemed to have a poorer prognosis (Figure 4M). On the other hand, we also validated whether the expression of tsRNA‐Thr‐5‐0096 is associated with ROS level. We reduced ROS levels using uric acid in A549 cells and performed qRT‐PCR. However, the result indicated that the expression of tsRNA‐Thr‐5‐0096 seemed not to be correlated with ROS level (Figure 4N).

### 3.5 The Effects of tsRNA‐Thr‐5‐0096 on Glycolysis and Oxidative Stress

**Figure 5:**
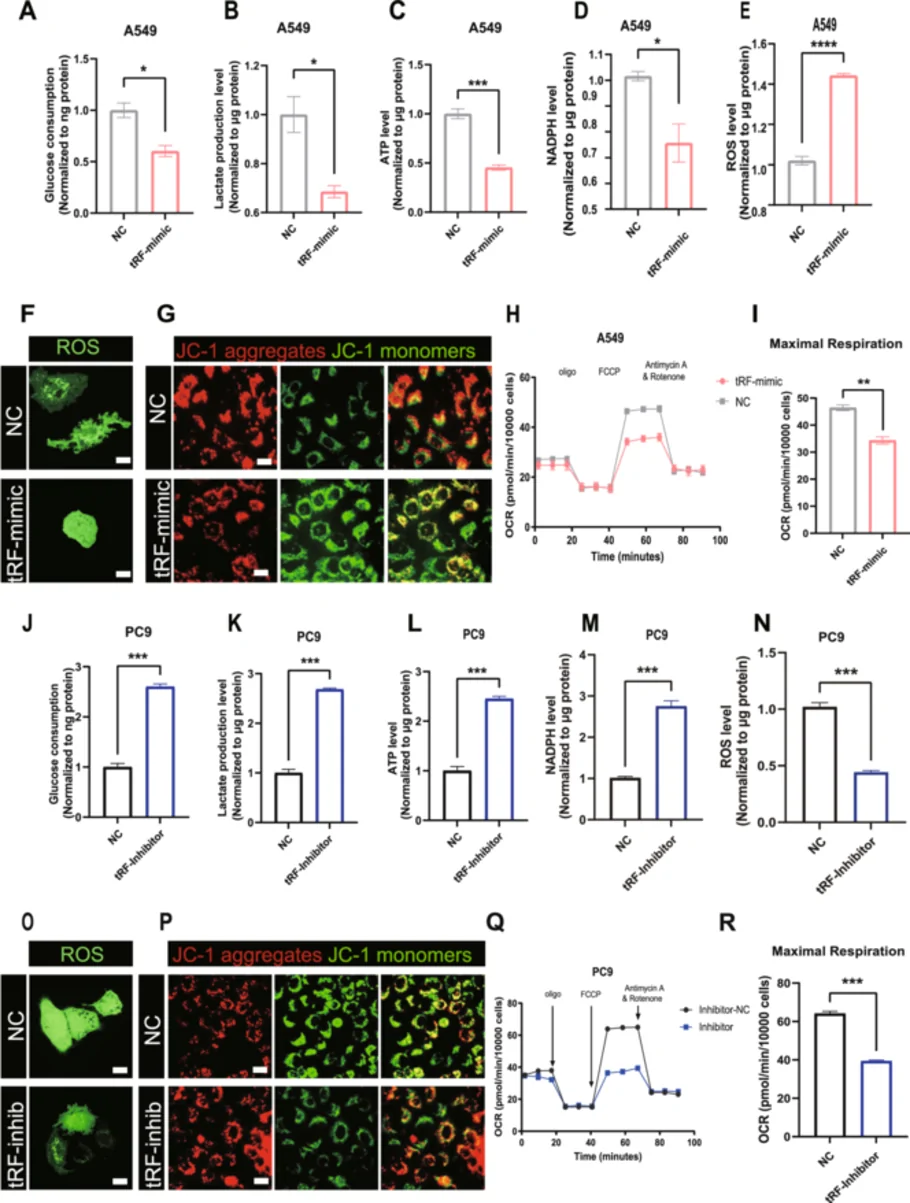
The effects of tsRNA‐Thr‐5‐0096 on glycolysis and oxidative stress. (A–C) Measurement of glucose uptake (A), lactate production (B), and ATP levels (C) in A549 cells transfected with tRF‐mimic or mimic‐NC. (D, E) Measurement of NADPH level (D) and ROS level (E) in A549 cells transfected with tRF‐mimic or mimic‐NC. (F) ROS staining on A549 cells transfected with tRF‐mimic or mimic‐NC, with a scale bar of 10 μm. (G) JC‐1 aggregates and JC‐1 monomers staining on A549 cells transfected with tRF‐mimic or mimic‐NC, with a scale bar of 10 μm. (H, I) OCR measurement in A549 cells transfected with tRF‐mimic or mimic‐NC. (J–L) Measurement of glucose uptake (J), lactate production (K), and ATP levels (L) in PC9 cells transfected with tRF‐inhibitor or inhibitor‐NC. (M, N) Measurement of NADPH level (M) and ROS level (N) in PC9cells transfected with tRF‐inhibitor or inhibitor‐NC. (O) ROS staining on PC9 cells transfected with tRF‐inhibitor or inhibitor‐NC, with a scale bar of 10 μm. (P) JC‐1 aggregates and JC‐1 monomers staining on PC9 cells transfected with tRF‐inhibitor or inhibitor‐NC, with a scale bar of 10 μm. (Q, R) OCR measurement in PC9 cells transfected with tRF‐inhibitor or inhibitor‐NC.

Given the evidence above, we validated the effect of tsRNA‐Thr‐5‐0096 on glycolysis and oxidative stress. As shown in Figure 5A–C, overexpression of tsRNA‐Thr‐5‐0096 inhibited glucose utilization, lactate concentrations, and ATP production in A549 cells. As expected, overexpression of tsRNA‐Thr‐5‐0096 reduced NADPH production while increasing ROS levels, with dysregulated mitochondrial function (Figure 5D–G). In addition, OCR experiments have demonstrated that overexpression of tsRNA‐Thr‐5‐0096 enhances maximal respiration in A549 cell lines (Figure 5H,I), indicating the opposite effect of tsRNA‐Thr‐5‐0096 on glycolysis and oxidative stress compared to G6PD. In contrast, we further validated the inhibition of tsRNA‐Thr‐5‐0096 in PC9 cells and obtained similar results (Figure 5J–R).

### 3.6 G6PD Mediated the Function of tsRNA‐Thr‐5‐0096 on LUAD Progression

**Figure 6:**
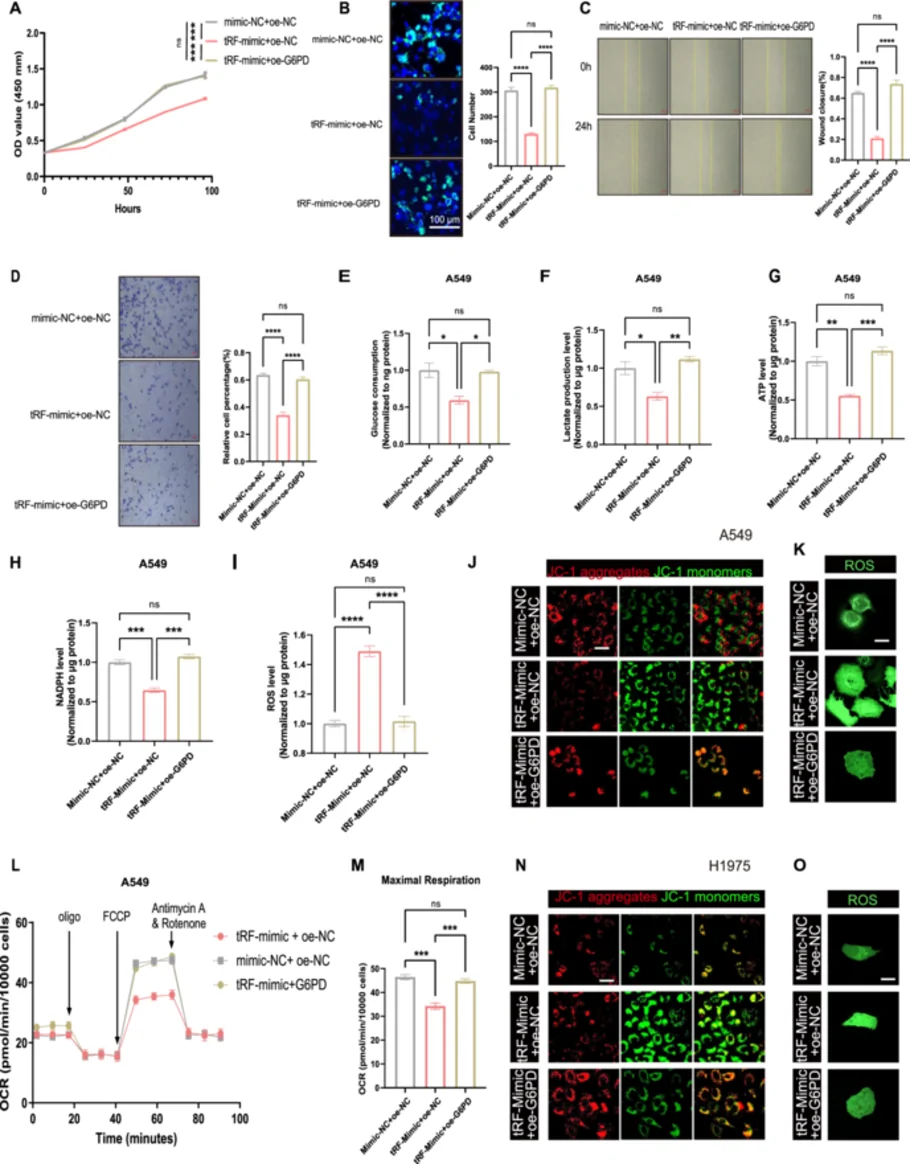
G6PD mediated the function of tsRNA‐Thr‐5‐0096 on LUAD progression. (A) CCK‐8 assays were conducted on A549 cells transfected with tRF‐mimic oe‐NC, mimic‐NC oe‐NC, or tRF‐mimic oe‐G6PD. (B) EdU assay was performed on A549 cells transfected with tRF‐mimic oe‐NC, mimic‐NC oe‐NC, or tRF‐mimic oe‐G6PD, with a scale bar of 100 μm. (C) A Transwell assay was conducted on A549 cells transfected with tRF‐mimic oe‐NC, mimic‐NC oe‐NC, or tRF‐mimic oe‐G6PD, with a scale bar of 50 μm. (D) Wound healing assay was performed on A549 cells transfected with tRF‐mimic oe‐NC, mimic‐NC oe‐NC, or tRF‐mimic oe‐G6PD, with a scale bar of 50 μm. (E–G) Measurement of glucose uptake (E), lactate production (F), and ATP levels (G) in A549 cells transfected with tRF‐mimic oe‐NC, mimic‐NC oe‐NC, or tRF‐mimic oe‐G6PD. (H, I) Measurement of NADPH level (H) and ROS level (I) in A549 cells transfected with tRF‐mimic oe‐NC, mimic‐NC oe‐NC, or tRF‐mimic oe‐G6PD. (J). JC‐1 aggregates and JC‐1 monomers staining on A549 cells transfected with tRF‐mimic oe‐NC, mimic‐NC oe‐NC, or tRF‐mimic oe‐G6PD, with a scale bar of 10 μm. (K). ROS staining on A549 cells transfected with tRF‐mimic oe‐NC, mimic‐NC oe‐NC, or tRF‐mimic oe‐G6PD, with a scale bar of 10 μm. (L, M) OCR measurement in H1975 cells transfected with tRF‐mimic oe‐NC, mimic‐NC oe‐NC, or tRF‐mimic oe‐G6PD. (N). JC‐1 aggregates and JC‐1 monomers staining on H1975 cells transfected with tRF‐mimic oe‐NC, mimic‐NC oe‐NC, or tRF‐mimic oe‐G6PD, with a scale bar of 10 μm. (O) ROS staining on H1975 cells transfected with tRF‐mimic oe‐NC, mimic‐NC oe‐NC, or tRF‐mimic oe‐G6PD, with a scale bar of 10 μm.

As mentioned above, tsRNA‐Thr‐5‐0096 binds to AGO2 protein and then targets the 3′UTR region of G6PD, leading to a decrease in its expression level. To confirm whether tsRNA‐Thr‐5‐0096 modulates LUAD cell progression, glycolysis, and oxidative stress through G6PD, the tsRNA‐Thr‐5‐0096 mimic and/or oe‐G6PD plasmid was transfected into A549 cells. EdU assays and CCK‐8 assays were performed, and it was identified that overexpression of tsRNA‐Thr‐5‐0096 significantly promoted cell proliferation of the LUAD cell lines; inhibition of overexpression of G6PD markedly weakened the functions of tsRNA‐Thr‐5‐0096 on LUAD cell proliferation (Figure 6A,B). In addition, wound healing and transwell assays suggested that overexpression of tsRNA‐Thr‐5‐0096 could significantly suppress LUAD cell migration and invasion, which could be weaken by G6PD overexpression (Figure 6C,D). To further confirm whether G6PD mediated the function of tsRNA‐Thr‐5‐0096 on glycolysis and oxidative stress, glucose utilization, lactate concentrations (Figure 6E–G), and ATP production, as well as NADPH production, ROS level, and mitochondrial function were performed (Figure 6H–O). The results indicated that tsRNA‐Thr‐5‐0096 regulates glycolysis and oxidative stress in LUAD cells through reducing G6PD expression. Collectively, G6PD mediated the function of tsRNA‐Thr‐5‐0096 on LUAD progression.

## 4 Discussion

In this study, we have successfully ascertained the presence of a tsRNA molecule associated with non‐invasive lung adenocarcinoma. Comprehensive in vitro functional assays have unequivocally demonstrated the potent inhibitory effects of this tsRNA on both the proliferation and metastasis of lung adenocarcinoma cell lines. Mechanistically, our research has revealed that the inhibition of G6PD mRNA expression by the tsRNA occurs through engagement in a miRNA‐like manner. This regulatory mechanism subsequently disrupts the intricate equilibrium between NADPH and ROS, thereby eliciting a profound inhibition of the malignant progression of lung adenocarcinoma. On the whole, our findings present a compelling case for the potential utilization of this tsRNA as an innovative and efficacious molecular target for the treatment of lung adenocarcinoma.

Metastasis remains a significant determinant of poor prognosis in lung adenocarcinoma. Non‐invasive LUAD is characterized by its slow growth and lack of significant changes during long‐term follow‐up, often resulting in a near 100% survival rate postoperatively. The prognosis for non‐invasive LUAD is favorable, with rare occurrences of recurrence and lymph node metastasis. In contrast, invasive LUAD is associated with a poorer prognosis, with a lower 5‐year survival rate. Understanding the differences between invasive and non‐invasive lung adenocarcinoma in terms of metastatic tendencies, diagnosis, and treatment approaches is crucial for developing personalized treatment strategies and improving patient outcomes. As a novel molecule, the function of tsRNA in lung adenocarcinoma is still poorly understood, especially in terms of metastasis. The application of tsRNA in the diagnosis and treatment of lung adenocarcinoma still has great prospects [9].

The mechanism of tsRNA regulation in cancer progression involves several types [11, 18, 19]. Firstly, tsRNA molecules are transcribed and processed from specific genomic loci or non‐coding RNA precursors. Subsequently, these tsRNAs interact with target genes or signaling pathways through various mechanisms [6, 20]. One common mechanism by which tsRNAs regulate cancer progression is through the inhibition of target gene expression [21, 22]. tsRNAs can bind to the mRNA of target genes, leading to their degradation or translational repression. This results in reduced expression of proteins involved in cell proliferation, invasion, and metastasis, thus inhibiting cancer progression [23]. For instance, tRFs have the potential to modify mRNA conformation, thereby affecting the translation process [24, 25]. For example, downregulation of 5′‐tiRNA^Val^ in serum was positively correlated with breast cancer stage progression and lymph node metastasis through inhibiting the frizzled class receptor 3(FZD3)‐mediated Wnt/β‐Catenin signaling pathway [13]. Further, Wang et al. reported that tsRNA could drive Aldolase A tetramer formation, thus resulting in tumor progression [26]. In the present study, we identified a tsRNA that inhibits the mRNA expression of G6PD through a type of miRNA‐like manner, thereby disrupting the balance between NADPH and ROS, leading to inhibition of the malignant progression of lung adenocarcinoma.

In conclusion, we identified a tsRNA, tsRNA‐Thr‐5‐0096, that inhibits the mRNA expression of G6PD through a type of miRNA‐like manner, thereby disrupting the balance between NADPH and ROS, leading to inhibition of the malignant progression of lung adenocarcinoma. Overall, this tsRNA may serve as a novel and effective therapeutic molecular target for lung adenocarcinoma.

## Author Contributions

**Suo Sha:** conceptualization, methodology, software, writing – original draft, writing – review and editing. **Meilin Zhang:** data curation, methodology, validation, supervision, investigation. **Qinsong Tao:** formal analysis, supervision, conceptualization. **Yang Liu:** funding acquisition, visualization, supervision, project administration, resources. **Junjun Bao:** resources, project administration, writing – original draft, writing – review and editing. **Ping Guo:** conceptualization, methodology, visualization, project administration, data curation. **Aiting Yan:** writing – original draft, writing – review and editing, conceptualization, methodology, software, data curation, formal analysis, resources. **Cuizhu Wang:** Conceptualization, investigation, funding acquisition, writing – original draft, writing – review and editing, resources.

## Supporting information


**Figure S1:** (A) Transwell assay was performed on A549 and PC9 cells, with a scale bar of 50 μm. (B) qRT‐PCR confirmed the transfection efficiency of oe‐G6PD. (C) CCK‐8 assays were conducted on A549 cells transfected with oe‐G6PD or oe‐NC. (D) EdU assay was performed on A549 cells transfected with oe‐G6PD or oe‐NC, with a scale bar of 100 μm. (E) Transwell assay was conducted on A549 cells transfected with oe‐G6PD or oe‐NC, with a scale bar of 50 μm. (F–H) Measurement of glucose uptake (F), lactate production (G), and ATP levels (H) in A549 cells transfected with oe‐G6PD or oe‐NC. (I, J) Measurement of NADPH level (I) and ROS level (J) in A549 cells transfected with oe‐G6PD or oe‐NC. (K, L) OCR measurement in A549 cells transfected with oe‐G6PD or oe‐NC

Supporting File

## Data Availability

The data that support the findings of this study are available on request from the corresponding author. The data are not publicly available due to privacy or ethical restrictions.
