# Supplementary material for: A tsRNA Suppresses Lung Adenocarcinoma Progression Through Targeting Glucose‐6‐Phosphate Dehydrogenase
Source: J Biochem Mol Toxicol. 2026 Sep 2;40(9):e71077. doi: 10.1002/jbt.71077 (PMC13535736; doi:10.1002/jbt.71077)
Supplement: Supplementary file 1 [file JBT-40-e71077-s001.pdf]

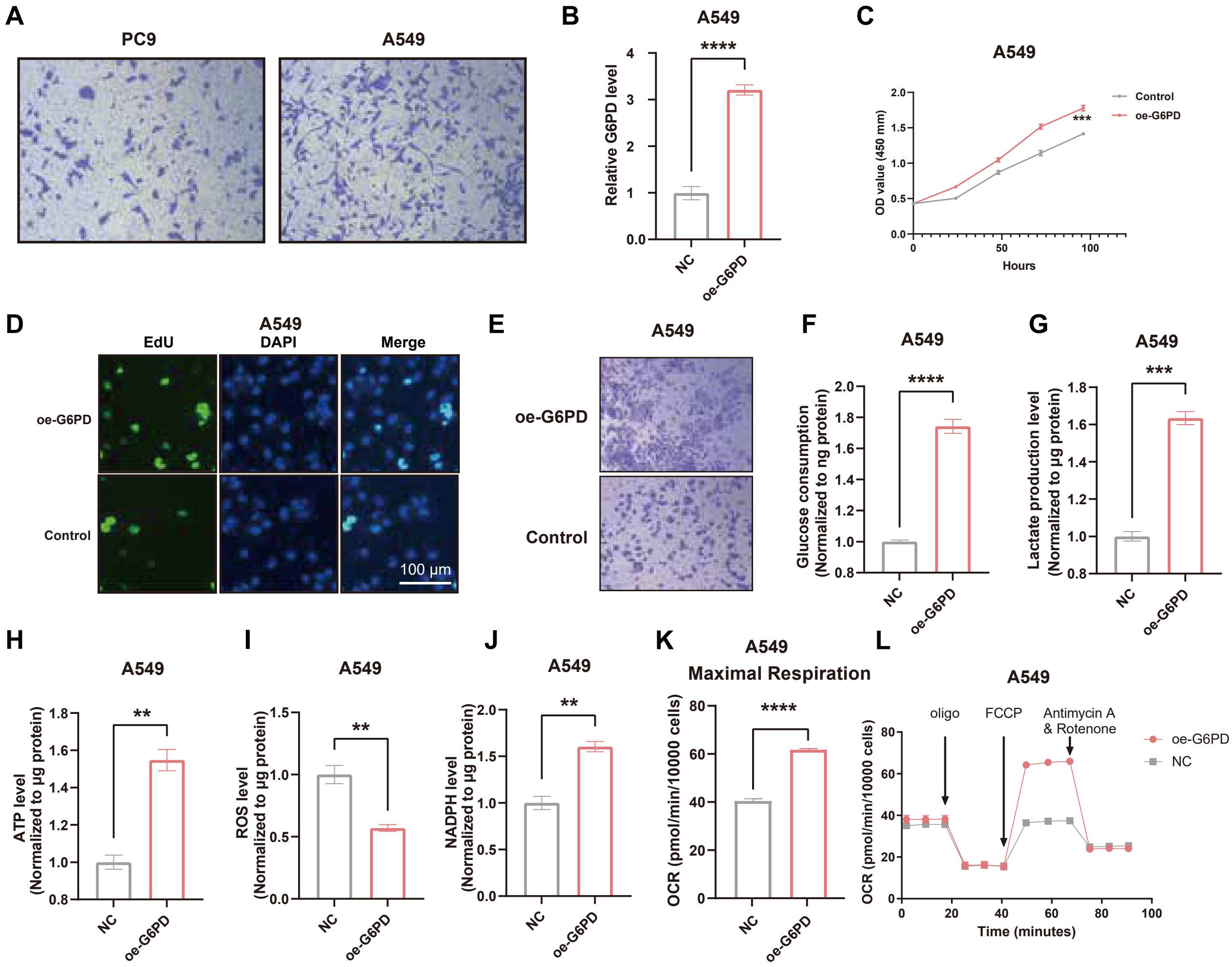
A
PC9
A549
B
A549
Relative G6PD level
****
NC
oe-G6PD
C
A549
OD value (450 nm)
Control
oe-G6PD
***
Hours
D
A549
EdU
DAPI
Merge
oe-G6PD
Control
100 μm
E
A549
oe-G6PD
Control
F
A549
Glucose consumption (Normalized to ng protein)
****
NC
oe-G6PD
G
A549
Lactate production level (Normalized to μg protein)
***
NC
oe-G6PD
H
A549
ATP level (Normalized to μg protein)
**
NC
oe-G6PD
I
A549
ROS level (Normalized to μg protein)
**
NC
oe-G6PD
J
A549
NADPH level (Normalized to μg protein)
**
NC
oe-G6PD
K
A549
Maximal Respiration
OCR (pmol/min/10000 cells)
****
NC
oe-G6PD
L
A549
oligo
FCCP
Antimycin A & Rotenone
oe-G6PD
NC
OCR (pmol/min/10000 cells)
Time (minutes)
