# Supplementary material for: A tsRNA Suppresses Lung Adenocarcinoma Progression Through Targeting Glucose‐6‐Phosphate Dehydrogenase
Source: J Biochem Mol Toxicol. 2026 Sep 2;40(9):e71077. doi: 10.1002/jbt.71077 (PMC13535736; doi:10.1002/jbt.71077)
Supplement: Supplementary file 2 [file JBT-40-e71077-s002.docx]

| **Table S1.** **Clinical information of patients involved in this study.** | |
| --- | --- |
| **Variable** | **Number of patients** |
|  |  |
| **Age** |  |
| ＜60 | 53 |
| ≥60 | 27 |
| **Sex** |  |
| female | 46 |
| male | 34 |
| **T-stage** |  |
| T1 | 45 |
| T2-T4 | 35 |
| **N-stage** |  |
| N0 | 53 |
| N1-N3 | 27 |
|  |  |

| **Table S2. The primer sequences and oligonucleotide sequences of the genes in this experiment.** | |
| --- | --- |
| **Oligo names** | **Sequences (5 ́-3 ́)** |
| FISH probe for tsRNA-Thr-5-0096 (labeled with cy3) | ACCCCUGAGCUAUGGAGCC |
| OE-G6PD | TGGGCCGGCGGGGCTCAGCCCCCGGAAACGGTCGTACACTTCGGGGCTGCGAGCGCGGAGGGCGACGACGACGAAGCGCAGACAGCGTCATGGCAGAGCAGGTGGCCCTGAGCCGGACCCAGGTGTGCGGGATCCTGCGGGAAGAGCTTTTCCAGGGCGATGCCTTCCATCAGTCGGATACACACATATTCATCATCATGGGTGCATCGGGTGACCTGGCCAAGAAGAAGATCTACCCCACCATCTGGTGGCTGTTCCGGGATGGCCTTCTGCCCGAAAACACCTTCATCGTGGGCTATGCCCGTTCCCGCCTCACAGTGGCTGACATCCGCAAACAGAGTGAGCCCTTCTTCAAGGCCACCCCAGAGGAGAAGCTCAAGCTGGAGGACTTCTTTGCCCGCAACTCCTATGTGGCTGGCCAGTACGATGATGCAGCCTCCTACCAGCGCCTCAACAGCCACATGAATGCCCTCCACCTGGGGTCACAGGCCAACCGCCTCTTCTACCTGGCCTTGCCCCCGACCGTCTACGAGGCCGTCACCAAGAACATTCACGAGTCCTGCATGAGCCAGATAGGCTGGAACCGCATCATCGTGGAGAAGCCCTTCGGGAGGGACCTGCAGAGCTCTGACCGGCTGTCCAACCACATCTCCTCCCTGTTCCGTGAGGACCAGATCTACCGCATCGACCACTACCTGGGCAAGGAGATGGTGCAGAACCTCATGGTGCTGAGATTTGCCAACAGGATCTTCGGCCCCATCTGGAACCGGGACAACATCGCCTGCGTTATCCTCACCTTCAAGGAGCCCTTTGGCACTGAGGGTCGCGGGGGCTATTTCGATGAATTTGGGATCATCCGGGACGTGATGCAGAACCACCTACTGCAGATGCTGTGTCTGGTGGCCATGGAGAAGCCCGCCTCCACCAACTCAGATGACGTCCGTGATGAGAAGGTCAAGGTGTTGAAATGCATCTCAGAGGTGCAGGCCAACAATGTGGTCCTGGGCCAGTACGTGGGGAACCCCGATGGAGAGGGCGAGGCCACCAAAGGGTACCTGGACGACCCCACGGTGCCCCGCGGGTCCACCACCGCCACTTTTGCAGCCGTCGTCCTCTATGTGGAGAATGAGAGGTGGGATGGGGTGCCCTTCATCCTGCGCTGCGGCAAGGCCCTGAACGAGCGCAAGGCCGAGGTGAGGCTGCAGTTCCATGATGTGGCCGGCGACATCTTCCACCAGCAGTGCAAGCGCAACGAGCTGGTGATCCGCGTGCAGCCCAACGAGGCCGTGTACACCAAGATGATGACCAAGAAGCCGGGCATGTTCTTCAACCCCGAGGAGTCGGAGCTGGACCTGACCTACGGCAACAGATACAAGAACGTGAAGCTCCCTGACGCCTATGAGCGCCTCATCCTGGACGTCTTCTGCGGGAGCCAGATGCACTTCGTGCGCAGCGACGAGCTCCGTGAGGCCTGGCGTATTTTCACCCCACTGCTGCACCAGATTGAGCTGGAGAAGCCCAAGCCCATCCCCTATATTTATGGCAGCCGAGGCCCCACGGAGGCAGACGAGCTGATGAAGAGAGTGGGTTTCCAGTATGAGGGCACCTACAAGTGGGTGAACCCCCA |
| The tsRNA-Thr-5-0096 mimic, mimic-negative control, tsRNA-Thr-5-0096 inhibitor, inhibitor-negative control and were designed and synthesized by Ribobio (Guangzhou, China). | |
